# Linking the Gut Microbial Ecosystem with the Environment: Does Gut Health Depend on Where We Live?

**DOI:** 10.3389/fmicb.2017.01935

**Published:** 2017-10-06

**Authors:** Nishat Tasnim, Nijiati Abulizi, Jason Pither, Miranda M. Hart, Deanna L. Gibson

**Affiliations:** Department of Biology, The Irving K. Barber School of Arts and Sciences, University of British Columbia, Kelowna, BC, Canada

**Keywords:** gut microbiome, immunity, environment, human health, immune tolerance, microbial colonization, biodiversity, microbe-rich environments

## Abstract

Global comparisons reveal a decrease in gut microbiota diversity attributed to Western diets, lifestyle practices such as caesarian section, antibiotic use and formula-feeding of infants, and sanitation of the living environment. While gut microbial diversity is decreasing, the prevalence of chronic inflammatory diseases such as inflammatory bowel disease, diabetes, obesity, allergies and asthma is on the rise in Westernized societies. Since the immune system development is influenced by microbial components, early microbial colonization may be a key factor in determining disease susceptibility patterns later in life. Evidence indicates that the gut microbiota is vertically transmitted from the mother and this affects offspring immunity. However, the role of the external environment in gut microbiome and immune development is poorly understood. Studies show that growing up in microbe-rich environments, such as traditional farms, can have protective health effects on children. These health-effects may be ablated due to changes in the human lifestyle, diet, living environment and environmental biodiversity as a result of urbanization. Importantly, if early-life exposure to environmental microbes increases gut microbiota diversity by influencing patterns of gut microbial assembly, then soil biodiversity loss due to land-use changes such as urbanization could be a public health threat. Here, we summarize key questions in environmental health research and discuss some of the challenges that have hindered progress toward a better understanding of the role of the environment on gut microbiome development.

## Introduction

Human health is closely linked to the diverse set of microorganisms in the intestine collectively known as the gut microbiota (Hooper and Gordon, 2001). This population of microorganisms and their genetic potential, or the gut microbiome, has been linked to human metabolism, intestinal homeostasis, immune development (Lynch and Pedersen, 2016), and brain processes and behavior (Mayer et al., 2015). A stable and diverse gut microbiota, optimal for maintaining health, produces metabolites that fuel physiological and metabolic processes. The gut microbiota also tunes local and systemic immune responses to confer protective immunity against pathogens while simultaneously maintaining immune tolerance toward commensals (Cerf-Bensussan and Gaboriau-Routhiau, 2010). Other functions of the gut microbiota include fermentation of indigestible dietary components (Flint et al., 2012), breakdown of environmental pollutants and pharmaceuticals (Claus et al., 2017), and pathogen competitive exclusion (Kamada et al., 2013). Alterations to the gut microbiota, known as dysbiosis, can disrupt these essential health-promoting services and are associated with gastrointestinal, cardiovascular, autoimmune and metabolic diseases (Carding et al., 2015). Therefore, the gut microbiome is a microbial ecosystem that operates much like a microbial organ that functions to promote health and prevent disease.

We are only beginning to understand the ecological processes that lead to the growth and development of a stable and diverse gut microbiome that promotes host-health. The gut microbiota is a diverse ecosystem comprised of bacteria, archaea, fungi and viruses including a diverse bacteriophage community (Manrique et al., 2016). Bacteria dominate the microbiota in abundance and diversity, with commensal members from seven phyla (Firmicutes, Bacteriodetes, Actinobacteria, Fusobacteria, Proteobacteria, Verrucomicrobia, and Cyanobacteria), the majority of which are uncultivated and novel phylotypes (Eckburg et al., 2005). Members of the microbiota can be permanent “residents,” transmitted through close contact between individuals, or transient “hitchhikers” from ingested food, water and various components of the environment (Ley et al., 2006; Harmsen and de Goffau, 2016). These transmission routes are important for establishing and maintaining microbial diversity in the gut (Browne et al., 2017). The mechanism of transmission can determine the pattern of colonization which shapes the gut microbial community of the host, but these patterns of transmission are poorly understood. Colonization that leads to the establishment of a stable and diverse adult gut microbiome lays the foundation for a homeostatic host-microbial relationship maintained by balanced immune responses. Colonizing gut microbes provide signals known as microbe-associated molecular patterns (MAMPs) that affect the maturation of the immune system and gut associated lymphoid tissue (GALT) (Wopereis et al., 2014). The development of the GALT is associated with bacterial activation of Toll-like receptors (TLRs) and downstream signaling pathways involved in maintaining host-microbial homeostasis, regulated through cytokines and chemokines (Hooper et al., 2015). Germ free animals have defects in the development of GALT, as well as cellular defects such as decrease in the number of lymphocytes, and molecular immune deficiencies such as reduced antibody production (Round and Mazmanian, 2009; Torrazza and Neu, 2011). Thus, colonization of the gut by microbes is not only important for the development of gut tissue, but also for the establishment of immune tolerance.

**Figure 1:**
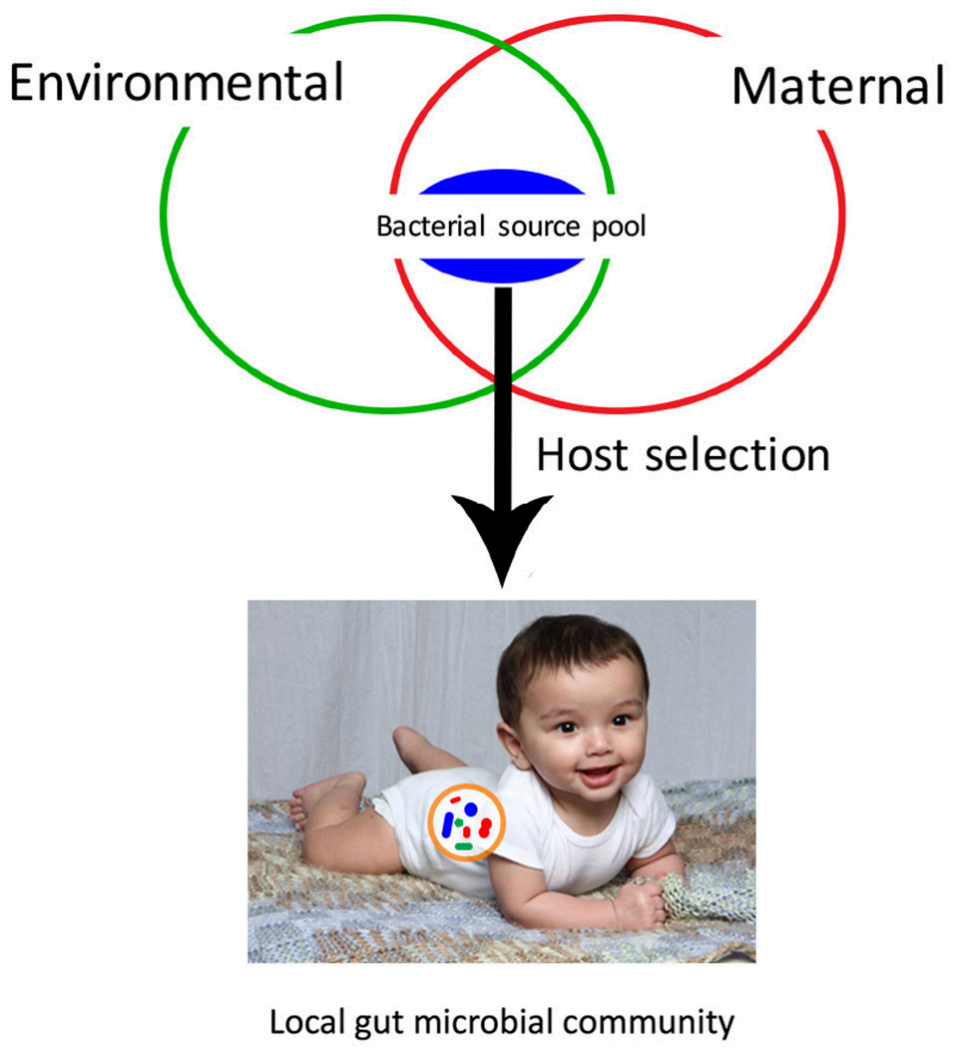
Local microbial community assembly of the infant gut microbiota depends on dispersal from a bacterial source pool. This bacterial source pool is comprised of both maternal microbes, transmitted vertically, and environmental microbes, transmitted horizontally. The development of the local community is shaped primarily by host selection, based on interactions between host and bacterial cells.

Gut bacterial community assembly begins pre-birth (Blaser and Dominguez-Bello, 2016), but rapid colonization takes place at birth and continues for the first 3 years of life (Lozupone et al., 2013). Two key factors that could influence the successful transmission of beneficial gut microbes to the infant are the mother and the external environment. Various studies that have sampled infant fecal microbiota have revealed that early gut microbial settlers that colonize the gut are derived from maternal vaginal, fecal, milk, mouth and skin microbiota during both gestation and birth through vertical transmission, and from the environment through horizontal transmission (Inoue and Ushida, 2003). Therefore, the infant gut microbiome is transmitted from a gut microbial species pool, comprised of gut symbionts from both the mother and the environment (Figure 1). The effect of the environment on the diversity and richness of the human gut bacterial species pool and gut microbiota transmission has yet to be explored. If the transmission of gut microbes is primarily parent-child, then environmental factors such as standards of hygiene, contamination of food and water by fecal microbes, delivery mode and hospitalization after birth can alter transmission mechanisms. On the other hand, if colonization patterns and gut microbiota diversity is linked to transmission of microbes from the external environment, then additional factors such as place of birth, geography, urban vs. rural living environment may also alter colonization of the gut microbiota affecting the human health. In this review, we discuss what is known about the role of environmental factors on the gut microbiota composition, diversity and assembly, identify major research challenges for research aiming to elucidate gut microbiota transmission patterns, and make suggestions for future studies that integrate the gut microbiome with environmental health research.

## Influence of environment on variations in gut microbiota diversity

The composition and diversity of gut microbiota varies between individuals. Under germ free conditions, gut microbiota transplantation experiments between model organisms such as zebrafish and mice have shown that gut microbiota composition is host-specific (Rawls et al., 2006). In humans, many other factors contribute to variation, such as diet, host genetics and metabolism, familial relationships, culture (Dominguez-Bello and Blaser, 2011), and demographics (Lozupone et al., 2013). According to global surveys of fecal microbiota from healthy populations, variation between individuals in richness of gut microbiota is largely explained by age, ethnicity (Huttenhower et al., 2012), geography (Torrazza and Neu, 2011), medication exposure, blood parameters, bowel, diet, health, anthropometrics and lifestyle (Falony et al., 2016). Of particular interest is the observation that healthy adults from rural societies such as Papua New Guinea (Martínez et al., 2015), Amerindia and Malawi (Clemente et al., 2015), and hunter-gatherers from Tanzania and Amazon (Schnorr et al., 2014) have higher gut bacterial species richness compared to urban populations in Italy and US. Similarly, children (between ages 1 to 5) from rural communities have more diverse gut microbiotas compared to children from Western populations (De Filippo et al., 2010). These host-specific differences in gut microbiota may arise from distinct selective pressures within the host gut habitat including genetics and diet but also may be due, at least in part, to their unique environments.

## Role of the environment on gut community assembly and immunoregulation

The role of the environment in the assembly of the gut microbiota has yet to be elucidated, although there is good reason to believe they are linked. Urbanization leads to changes in living conditions such as increased sanitation and antibiotic use (Popkin, 1999), separation from the outdoors (Turner et al., 2004), and poor land management practices that may reduce soil microbial biodiversity (Wall et al., 2015). Accordingly, studies show that infants born via caesarian section have altered colonization patterns and lower total gut microbiota diversity (Biasucci et al., 2010), and individuals who grow up in city environments have a less diverse gut microbiome (Sjögren et al., 2009). Further, urbanites are more prone to inflammatory disorders like diabetes and multiple sclerosis (Kay, 2000) as well as allergic diseases such as asthma (Rook, 2012) during both infancy and adulthood (Garn and Renz, 2007). Although host genetics may in large part determine the composition of the adult gut microbiome, it has been shown that alien microbes from diverse habitats like soil can colonize the germ-free gut (Seedorf et al., 2014). Therefore, horizontal transmission of environmental microbes may be contributing commensal microbes to the gut ecosystem, altering patterns of colonization to increase variation in gut microbiota diversity.

Early-life exposure to microbe-rich environments may be beneficial for human health by increasing the gut bacterial species pool. The “microbial old friends” hypothesis, posits microbe-rich environments are a source of beneficial microbes that promote gut microbiota diversity (Zhou et al., 2015) reducing inflammatory disease risk (Rook et al., 2013). Indeed, growing up in microbe-rich environments, like traditional farms, result in healthier children (Mosca et al., 2016). Therefore, the prevalence of inflammatory disorders may be higher in modern cities because of reduced exposure to beneficial microbes from the environment, such as microbes from house dust or zoonotic microbes from animals. Indeed, exposure to household pets has been shown to alter the infant gut microbiota and reduce allergic disease (Tun et al., 2017). Reduced exposure to pathogenic microorganisms, largely as a result of modern hygienic practices, can also result in defective immunoregulation (Garn and Renz, 2007). The “hygiene hypothesis” makes the argument that infectious stressors are particularly important during early childhood (Wills-karp et al., 2001; Garn and Renz, 2007) and is supported by epidemiological studies showing rural children have reduced asthma (Ege et al., 2011), hay fever (Strachan, 1989) and ectopic eczema (Isolauri et al., 2000). Such allergic diseases are chronic inflammatory disorders caused by a decrease in immune tolerance (Garn and Renz, 2007). Decrease in tolerance is associated with a decrease in Treg cells expressing the transcription factor forkhead box P3 (FOXP3+ Treg cells) (Simon et al., 2015). FOXP3+ Treg cells produce anti-inflammatory cytokines such as interleukin 10 (IL-10) and transforming growth factor-β (TGF-β) which help to suppress exacerbating inflammatory responses and balance CD4+ helper T (Th) Th1 and Th2 cells. In allergic diseases, cytokine stimulation of naïve T cells from IL-4, IL-5 and IL-13 tilt the balance of Th cells toward the Th2 phenotype (Kay, 2000). In infants, there may be a normal Th2 bias observed in both mice (1–3 weeks old) and humans (0–2 years old) (Marchant and Goldman, 2005; Dowling and Levy, 2014). As the infant ages, the Th2 skew is balanced by Th1 responses and induced memory responses through mucosal-associated invariant T cells and interleukin-8 (CXCL8) secreting naïve T cells (Simon et al., 2015). In contrast, allergic infants have a persistent Th2 phenotype, resulting in long term Th2-skewed immunity (Barrios et al., 1996). Therefore, early life exposure to a broad range of immunoregulation-inducing commensal and pathogenic environmental microorganisms can provide a Th1 stimulus, conferring protection against immune disorders.

What is it about urban environments that reduces healthy gut microbiome functioning? Both “old friends” and the “hygiene hypothesis,” are contingent on microbial biodiversity. Urban development leading to the loss of local habitats and biodiversity may be detrimental to human health by depleting or otherwise altering the reservoirs of environmental microbes including bacteria, fungi and viruses that may play a role in gut microbiota-mediated immune health. The “biodiversity hypothesis” posits that clinical diseases, caused by poor microbiome, immune dysfunction and inflammation, are linked to biodiversity loss (Anderson et al., 2013). Biodiversity loss due to industrialization is associated with adverse health effects, including inflammatory diseases (Haahtela et al., 2013). Environmental biodiversity and immune function have been linked in epidemiological studies, which show individuals living in built environments have lower diversity of microbiota and higher allergic disposition (Wardle et al., 2004). The World Allergy Organization has proposed that loss of biodiversity is linked to loss of microbial diversity, resulting in microbial deprivation and ultimately, inflammatory disorders (Haahtela et al., 2013). This proposal extends the “old friends” and hygiene hypothesis to include environmental biodiversity as being important in the development of the immune system and gut microbiome (von Hertzen et al., 2011). A biodiverse environment that is microbe-rich may promote the development of healthy gut microbiota and lower disease risk.

Extending the “biodiversity hypothesis” to include soil biodiversity has the potential to provide more insight into the role of the environment and gut mediated immune health. Soils contain a dynamic reservoir of biodiversity (Torsvik and Øvreås, 2002) and this diversity is essential for maintaining biogeochemical processes and ecosystem functioning (Wardle et al., 2004). In this way, soil biodiversity provides benefits to human health indirectly through suppression of soil-borne pathogens, provision of clean air, water and food, and exposure to immunoregulation-inducing soil microorganisms (Wall et al., 2015). Although unknown, we ask if there is a direct link between soil microbial diversity and human health? Certainly, soil microbial diversity varies in taxonomic composition between biomes (Fierer et al., 2012b), physical and chemical gradients (Fierer et al., 2012a; Lauber et al., 2013), and anthropogenic activity (Ramirez et al., 2010). Whether it is species richness that is important, or the composition of key taxa has not been determined. There is some indirect evidence that soil biodiversity and human microbiota are interrelated (Hanski et al., 2012), to provide “natural immunity” (von Hertzen et al., 2011). Further, exposure to soil microbes has been experimentally shown to increase gut microbiota diversity (Zhou et al., 2015). There is also some evidence to suggest that exposure to possible soil pathogens could contribute to immune tolerance (Wall et al., 2015). However, little is known about the impact of soil exposure on gut microbiota transmission and colonization patterns in humans.

## Exploration of the connections between soil microbial communities and gut microbial communities

**Figure 2:**
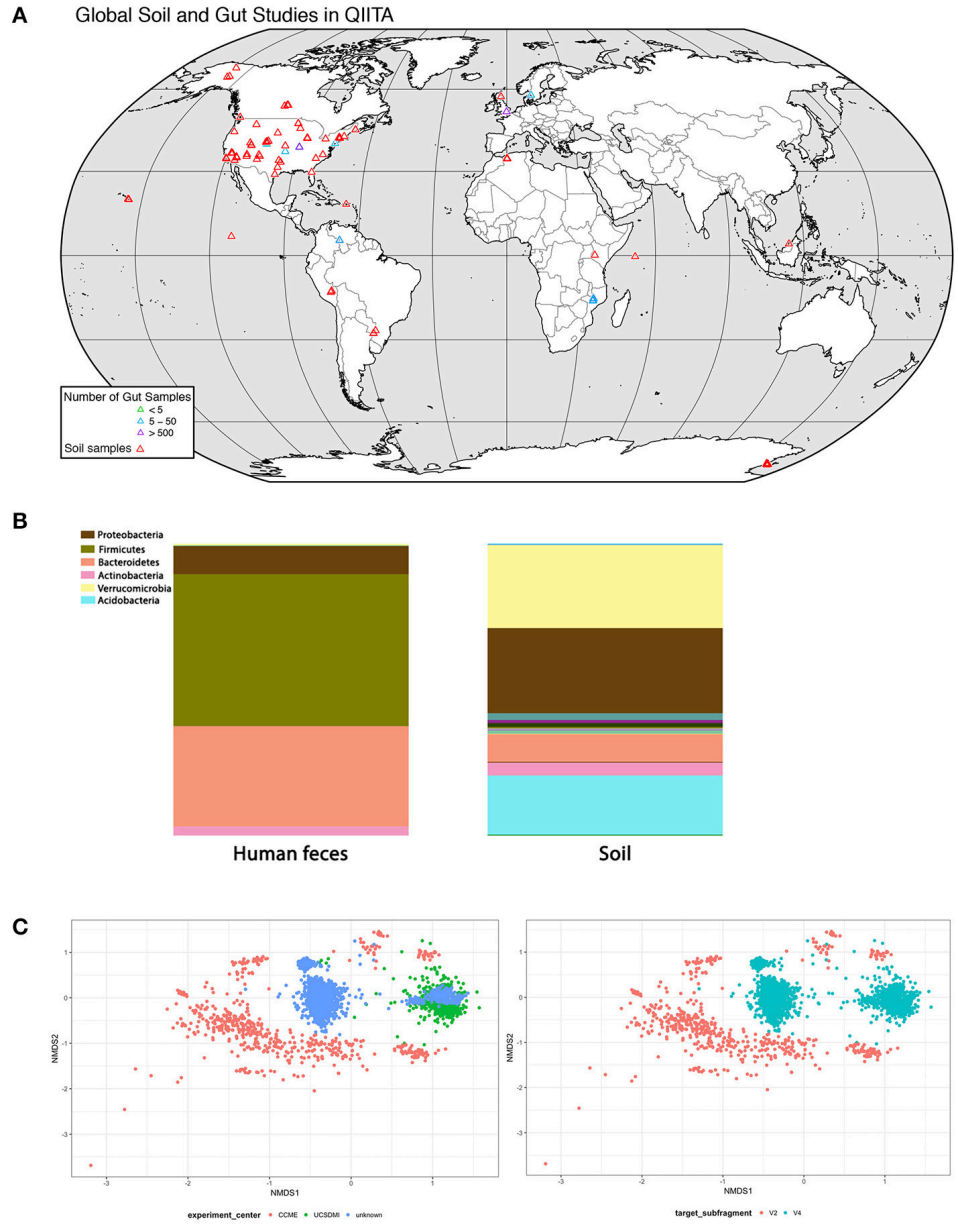
Analysis of concurrence between gut and soil microbiome studies (data deposited in http://qiita.microbio.me) **(A)** Geographical range of soil (red symbols) and gut studies (green, blue, and purple symbols) available on QIITA. Studies are predominantly located in North America and Europe. Human fecal samples were collected from 14 countries, but the vast majority (66%) are from USA, Malawi, and Venezuela. Samples were collected from a range of ages (0–77 years). Soil samples were taken from a variety of habitats, including wetlands, garden soils, tundra, and tropical biomes. **(B)** Relative proportion of bacterial phyla in human feces (*n* = 2,497) and soil (*n* = 1,123) samples combined from seven gut and four soil studies in QIITA show little overlap of bacterial taxa. **(C)** Non-metric multidimensional scaling (NMDS) ordination plot of Bray-Curtis community dissimilarities on OTUs from 16 s gene sequences from four US gut studies conducted by the same principal investigator (Rob Knight) (2D stress value = 0.16). Samples show clustering according to study center (*n* = 935) (plot on left) as well as primer choice (plot on right) where V2 subfragment (*n* = 35) or V4 subfragment (*n* = 900) is targeted. Symbols represent individual fecal samples.

For soil biodiversity to be relevant to human health requires microbes from local soil to be transmitted horizontally to humans and then established in the gut. If so, then people exposed to similar soil microbial communities should have more similar gut communities. We analyzed soil and gut studies from publicly available datasets on Qiita (http://qiita.microbio.me), to investigate the link between soil and gut microbial diversity, in terms of richness, diversity and species identity. We combined OTU (operational taxonomic unit) tables from 7 gut studies (*n* = 2,497 human fecal samples) and 4 soil studies (*n* = 1,123 soil samples) that used 16 s amplicon sequencing to study bacterial communities (Figures 2A,B). The human fecal samples were collected from 14 countries, although the vast majority of samples were from USA (*n* = 1,062), Malawi (*n* = 1,042) and Venezuela (*n* = 99). These samples were collected from a range of ages (0–77 years). A small proportion of adult humans were diagnosed with obesity, atherosclerosis (*n* = 52). Soil studies were from 17 different locations and most samples were from North America (*n* = 1062). Soil samples ranged from wetland to garden soil from tundra to tropical biomes.

### US studies dominate

Although our dataset was diverse, we lacked sufficient data to explore global variation in soil-gut microbiota. At the time of analysis, there were 244 studies on Qiita of which we picked four large-scale soil and seven gut studies to pool into a combined dataset (Figure 2A). Most studies on soil and human bacterial communities were located in the US. Future efforts should survey populations from different countries and physiographic regions to provide global geographical gut microbial datasets.

### There is little overlap between soil and gut microbes at lower taxonomic levels

We performed downstream analysis to compare the relative proportion of bacterial phyla in human gut and soil samples (Figure 2B). We visualized the OTUs in human gut and soil samples using taxa summary plots. Samples were grouped and averaged by sample type (gut or soil) and taxonomic composition was summarized on multiple taxonomic levels (e.g., phylum, order, etc.) (Navas-Molina et al., 2013). We found that human fecal samples were dominated by Bacteriodetes and Firmicutes phyla, whereas soil samples were dominated by Proteobacteria and Verrucomicrobia. These differences in taxonomic composition between soil and gut samples were also consistent at lower taxonomic levels (see Figure 2B table).

### Study effects account for variation

Differences in DNA extraction protocol, primer selection, sequencing platform and sequence analysis pipelines introduce bias to datasets known as study effects. To evaluate the influence of study effects, we pooled all human gut studies from a single investigator (Rob Knight, University of California), and excluded all studies outside the US resulting in four studies. We tested for study effects (*n* = 935) by considering research group (Figure 2C) and primer subfragment (Figure 2C). We found that primer target region or research group contributed to strong study-based clustering, similar to clustering patterns found in other meta-analyses of the human microbiota (Lozupone et al., 2013). Our results indicate that soil and gut bacterial communities have few overlapping taxa, but because most gut and soil studies survey North American cohorts, we were not able to determine whether local soil microbial communities influence the composition of gut microbial communities of individuals from different geographical locations.

## Challenges for studying environment-gut microbiota interactions in health and disease

Global surveys on the relationships between environment, gut microbiota and inflammation are yet to be explored, such as how traditional diets consumed in a region may contribute to the gut microbial community or how local soil influences diversity of the gut microbiota of the population through horizontal transmission. The mechanism of horizontal transmission of environmental microbes, whether inhalation, ingestion or cutaneous, also remains to be elucidated. The rate of urbanization and soil degradation may be related to changes in the composition of the gut microbiota, such as an increase abundance of bacterial indicators of dysbiosis such as Proteobacteria (Shin et al., 2015). We cannot begin to understand the link between soil microbial diversity and gut microbial assembly until studies adopt standardized collection, extraction and sample preparation procedures with complete and transparent metadata reporting and appropriate analysis platforms. Some additional challenges for study design and analysis of environment-gut studies are outlined below.

### Challenge 1: quantifying microbe-richness and diversity of the environment

To link gut microbiota to environmental microbial diversity, it will be important for future studies to develop standardized methods that reliably reflect microbial biodiversity in the environment. In addition to microbial diversity of the direct environment (home, air, soil, water, etc.), biodiversity of the surrounding environment should be estimated by recording information about the landscape, including land use type and predominant vegetation structure, abiotic factors such as climatic factors and information about the biodiversity of resident communities (i.e., plants, animals, etc.; as described by Hanski et al., 2012). Given the logistical challenges associated with such efforts, we recommend choosing sampling locations strategically in relation to desired environmental (Metzger et al., 2013) and other characteristics. This approach will help elucidate associations between microbial exposure, environmental biodiversity, and gut microbiota assembly.

Together, these parameters will help elucidate direct effects of microbial exposure and environmental biodiversity on gut microbiota assembly.

### Challenge 2: collection, storage and analysis of host-microbiome-environment interactions

Developing analysis tools and platforms that are able to store and analyze large datasets will be critical to link gut microbiota assembly to external factors. Currently, limitations in sample collection, processing and storage (Gorzelak et al., 2015), as well as systems of reporting, study design, sample size, variation in demographics and statistical approaches prevent cross study comparisons (Hunter, 2005). The two publicly-available platforms for microbiome-environment studies are Qiita and SourceTracker (Knights et al., 2011), yet these have had little uptake by the community as a whole. To fully understand demographic factors in gut microbial assembly, this will need to be a globally coordinated effort. The utility of the NIH Human Microbiome Project (http://www.hmpdacc.org/) could be enhanced by including protocols and repositories for environmental biodiversity (microbial and otherwise). Applications such as SourceTracker could then be easily used to investigate source-sink dynamics of the microbiota, to investigate microbiome-exposure interactions on the ecology of the microbiome. Once the challenge of data collection, handling and analysis are met, microbiome changes can be used as biomarkers to indicate individual health and disease outcome (Segata et al., 2011).

## Conclusion

The study of environmental influences on gut microbiota structure and function is especially pertinent because the human living environment is becoming rapidly urbanized. Such drastic changes to the human environment may interrupt the healthy development of the microbiota and increase risk of inflammatory diseases. Moving forward, we must incorporate gut microbiota surveys into a broader framework of environmental exposure, for a thorough understanding of how ecosystem processes contribute to gut microbiota development, and affect the quality of human health.

## Author contributions

NT: data collection and analysis; statistical analysis; intellectual design; writing and editing of the manuscript. NA: data collection and analysis; statistical analysis. JP: data analysis; statistical analysis oversight; editing of the manuscript. MH: data analysis; intellectual design; writing and editing of the manuscript; funding support. DG: intellectual design; writing and editing of the manuscript; funding support.

### Conflict of interest statement

The authors declare that the research was conducted in the absence of any commercial or financial relationships that could be construed as a potential conflict of interest.
